# Sequence-Dependent Antiproliferative Effects of Gefitinib and Docetaxel on Non-Small Cell Lung Cancer (NSCLC) Cells and the Possible Mechanism

**DOI:** 10.1371/journal.pone.0114074

**Published:** 2014-12-04

**Authors:** Bei Chen, Jingxian Zheng, Yunyun Zeng, Baofeng Li, Bo Xie, Jihua Zheng, Juan Zhou, Weimin Zhang

**Affiliations:** Department of Oncology, Guangzhou General Hospital of Guangzhou Military Command, Guangzhou, Guangdong, China; University of Central Florida, United States of America

## Abstract

**Purpose:**

Recent clinical trials showed that the sequential combination of epidermal growth factor receptor tyrosine kinase inhibitors (EGFR-TKIs) and chemotherapy could prolong the PFS and/or OS of advanced non-small cell lung cancer (NSCLC) patients with EGFR mutation. The aim of present study was to assess the optimal combination sequence and to explore its possible mechanism.

**Methods:**

PC-9 cells and A549 cells, the lung adenocarcinoma cells with mutant and wide-type EGFR respectively, were treated with docetaxel/gefitinib alone or in different combination schedules. The EGFR and K-ras gene status was determined by qPCR-HRM technique. Cell proliferation was detected by MTT assay. The expression and phosphorylation of EGFR, ERK, Akt and IGF-1R were detected by western blot. Cell cycle distribution was observed by flow cytometry.

**Results:**

Only sequential administration of docetaxel followed by gefitinib (D→G) induced significant synergistic effect in both cell lines (Combination Index<0.9). The reverse sequence (G→D) resulted in an antagonistic interaction in both cell lines (CI>1.1), whereas the concurrent administration (D+G) showed additive (0.9<CI<1.1)-synergistic effect in PC-9 cells and antagonistic-additive effect in A549 cells. Mechanism studies showed that docetaxel-induced phosphorylation of EGFR and ERK was repressed by subsequently used gefitinib, but not by concurrent exposure of gefitinib. The gefitinib-repressed phosphorylation of EGFR and ERK was reversed neither by concurrent nor by subsequent administration of docetaxel. D+G reinforced their inhibition on the phosphorylation of IGF-1R in PC-9 cells.

**Conclusions:**

The cytotoxic drugs followed by EGFR-TKIs may be the optimal combination for antiproliferative effects in EGFR-mutant NSCLC cells, and the phosphorylation of EGFR and ERK might contribute to this effect.

## Introduction

Non-small cell lung cancer (NSCLC) is the leading cause of cancer death worldwide. It is well known that for treatment of advanced NSCLC, epidermal growth factor receptor tyrosine kinase inhibitor (EGFR-TKI) and chemotherapy is recommended as first-line therapy for patients with active EGFR mutation and wild type EGFR, respectively. This recommendation is based on the results of a phase III randomized trial IPASS in which patients with EGFR mutations who received gefitinib had increased progression-free survival (PFS 24.9% vs. 6.7%), response rate (RR 71.2% vs. 47.3%) and quality of life when compared with those receiving chemotherapy [1]. However the application of platinum-based chemotherapy and EGFR-TKI has reached a therapeutic plateau. Although no new revision appears in the last guideline, some phase III clinical trials including FASTACT-2 [2] and INFORM [3] have taken a further step and showed that chemotherapy combined with EGFR-TKI in specific schedules could improve the prognosis, especially in patients with EGFR mutations. Accordingly, we presumed that further improvements might come from the findings of new target, the overcoming of EGFR-TKIs tolerance and the combination of EGFR-TKI with chemotherapy since the mechanisms of their anti-tumor activity are different.

For the combination treatment, basically three schedules were discussed in recent clinical trials: 1. concurrent administration; 2. chemotherapy followed by EGFR-TKI; 3. EGFR-TKI followed by chemotherapy. INTACT-2, TRIBUTE and TALENT studies showed that response rate and overall survival (OS) favored concurrent combination only in EGFR-mutant patients, but not in wild-type or unselected patients [4]–[6]. WJTOG0203 and INFORM trials demonstrated that sequential administration of chemotherapy followed by EGFR-TKI seemed beneficial for unselected population (with significantly improved OS and PFS) [7], [3]. Another phase III study FASTACT-2 recently reported that intercalated chemotherapy and erlotinib was another viable first-line option for patients with unknown EGFR status. It was shown that PFS and OS were significantly prolonged with chemotherapy plus erlotinib vs. chemotherapy plus placebo (PFS: 7.6 m vs. 6.0 m, P<0.0001; OS: 18.3 m vs. 15.2 m, P = 0.042). The benefit was even greatest for EGFR-mutant patients [2]. By contrast, Kanda et al showed in a phase II trial that gefitinib followed by chemotherapy gained a better PFS in EGFR-mutant patients compared with previous reports using gefitinib alone as the first-line treatment [8]. However, by now no clinical trial has compared the three schedules with each other and told which one was optimal. In this regard, the first aim of the present study is to find out the optimal schedule from three different combination strategies of docetaxel and gefitinib.

On the other hand, the cellular mechanism of sequence-dependent effect of gefitinib in combination with chemotherapeutic agents remains an open question. Some previous studies indicated that the synergistic effect induced by sequentially administered EGFR-TKI following cytotoxic agents might be correlated with EGFR phosphorylation, whereas an antagonistic effect of EGFR-TKIs followed by chemotherapy was related with the regulation of cell cycle distribution [9], [10]. Moreover, recent investigations reported that insulin-like growth factor receptor-1 (IGF-1R) affected the cell sensitivity to chemotherapy as well as EGFR-TKIs through the regulation of signaling pathway such as PI3K/AKT and MARK/ERK [11], [12]. Basing on these findings, our second aim is to probe into the cellular mechanism of sequence-dependent effects of docetaxel and gefitinib on lung cancer cells through assessment of the possible changes in the above signaling pathways and in cell cycle distribution.

## Materials and Methods

### Materials

Gefitinib (Astra Zeneca, UK) and docetaxel (Sanofi Aventis, France) were prepared in dimethyl sulfoxide (DMSO) (Sigma Aldrich, USA) and stored at –20°C. The drugs were diluted in fresh media before the experiment, and the final DMSO concentration never exceeded 0.1%.

### Cell Culture

Human lung adenocarcinoma cell A549 and PC-9 [13] were kindly given by Institute of Biochemistry and Cell Biology (Shanghai, China) and National Cancer Center Hospital of Japan (Tokyo, Japan), respectively. The study was approved by the ethics committee and review board of our hospital. Cells were cultured in RPMI-1640 medium supplemented with 10% bovine serum (Gibco, Grand Island, NY, USA) in a humidified atmosphere (Forma Scientific, Marietta, OH, USA) containing 5% CO_2_ at 37°C.

### Identification of EGFR and K-ras gene status

DNA was extracted from A549 and PC-9 cells using the QIAmp tissue kit (Qiagen, Hilden, Germany) according to the manufacturer’s instructions. Exons 18, 19, 20 and 21 of EGFR gene as well as exons 2 and 3 of K-ras gene were detected using quantitative PCR high-resolution melting (qPCR-HRM) technique.

### MTT assay

A549 and PC-9 cells were seeded in 96-well plates (10^4^ cells per well) and were divided into 6 groups and treated as follows: 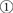 192 Control group (C): cells were incubated with PBS for 72 h; 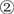 193 Docetaxel group (D): cells were treated with docetaxel for 72 h; 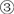 194 Gefitinib group (G): cells were treated with gefitinib for 72 h; 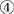 Docetaxel→Gefitinib group (D→G): cells were incubated with docetaxel for 24 h followed by gefitinib for 48 h; 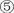 Gefitinib→Docetaxel group (G→D): cells were incubated with gefitinib for 48 h followed by docetaxel for 24 h; 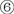 Concurrent group (D+G): cells were incubated concurrently with docetaxel and gefitinib for 72 h. Cell proliferation was determined by MTT assay. The optical density (OD) at 490 nm was determined using a 96-well multiscanner auto-reader (Dynatech MR 5000). Each test was performed in triplicate. Inhibition rate% = 100%–(OD _test_–OD _blank_)/(OD _control_–OD _blank_)×100%. Each experiment was repeated in triplicate.

### Calculation of Combination Index (CI)

The antiproliferative effects of the combined treatment were evaluated by Combination Index (CI). Cells were treated with three different sequences as mentioned above: 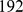 D→G; 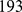 G→D; 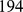 D+G. The drug doses were combined using constant ratios of IC50 values calculated from MTT assay. Thus, we used 0.125, 0.25, 0.5, 1, 2 and 4 times of IC50 doses of docetaxel and gefitinib. The results of sequential treatments were analyzed according to the method of Chou [14]. The CI value was calculated using CompuSyn software (ComboSyn, Inc., Paramus, NJ, USA), with CI>1.1, CI = 0.9–1.1 and CI<0.9 indicating antagonistic, additive and synergistic effects, respectively. Each experiment was repeated in triplicate.

### Western blot

A549 and PC-9 cells were lysed in buffer containing proteinase inhibitors. The total protein of cells was obtained using the Nuclear Extract Kit (Active Motif Corp, USA). Protein concentration was determined by the BCA protein assay (Pierce, Rockford, IL, USA). Samples containing 100 µg of total protein were electrophoresed on 10% SDS-PAGE and transferred onto a nitrocellulose membrane by electroblotting. The blots were probed using the following antibodies: anti-EGFR (1∶1000; Santa Cruz, CA), anti-phosphorylated-EGFR (1∶1000), anti-ERK1/2 (1∶600), anti-phosphorylated-ERK1/2 (1∶600), anti-AKT (1∶1000), anti-phosphorylated-AKT (1∶1000), anti-IGF-1R (1∶600), anti-phosphorilated-IGF-1R (1∶600) and anti-β-actin antibody (1∶800) (all the above antibodies were from Cell Signaling Technology, USA), followed by incubation with horseradish peroxidase (HRP) conjugated goat-anti-mouse secondary antibody (Santa Cruz, CA, USA). The blots were visualized by an enhanced chemiluminescence kit (ECL) (Amersham Pharmacia Biotech, Arlington Heights, IL, USA) according to the manufacturer’s instructions. Each experiment was performed in triplicate.

### Cell cycle analysis

Freshly prepared cells were transferred to cryopreservation tubes. Each group consisted of 3 tubes. After treatments, cells were washed twice with PBS and then fixed with 70% alcohol at 4°C overnight. After centrifugation at 1000 rpm for 5 min, cells were incubated with propidium iodide (Sigma-Aldrich, St. Louis, MO, USA) at 4°C for 30 min in the dark before being subjected to a flow cytometer (FACScan, Becton Dickinson, Mountain View, CA). Cell cycle was analyzed using Multicycle-DNA Cell Cycle Analyzed software. Each experiment was performed in triplicate.

### Statistical analysis

The differences between means were analyzed with ANOVA and data were expressed as mean ± *SD*. All statistical analyses were performed using SPSS 13.0 software (Chicago, IL). Differences were considered statistically significant when *P*<0.05.

## Results

### EGFR and K-ras gene status in A549 and PC-9 cell lines

qPCR-HRM technique showed that A549 cells harbored a mutation in K-ras exons 2 and no mutation in EGFR gene. PC-9 cells harbored a mutation in EGFR exons 19 and no mutation in K-ras, which was in accordance with previous studies [13].

### Effects of different exposure schedules of gefitinib and docetaxel on cell proliferation

**Figure 1:**
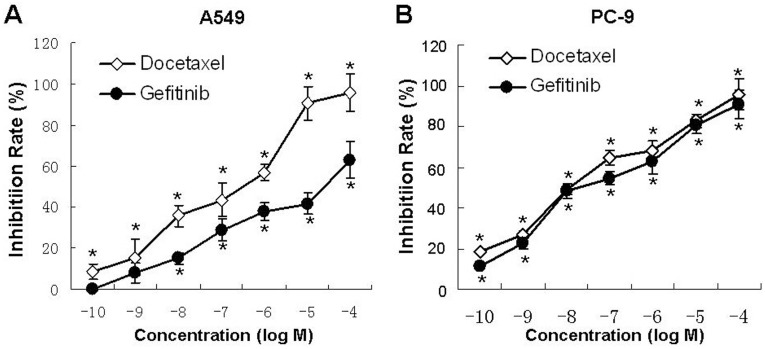
Docetaxel or gefitinib alone inhibited the proliferation of lung adenocarcinoma cells in a dose-dependent manner. Cells were treated with gradient concentrations (10^−4^ M∼10^−10^ M) of gefitinib or docetaxel for 72 h. Cell proliferation was determined by MTT assay. (A) A549 cells; (B) PC-9 cells. *P<0.05 compared with control group. *Bars*: ± SD, n = 3.

MTT assay showed that docetaxel (10^−4^ M∼10^−10^ M) or gefitinib (10^−4^ M∼10^−8^ M for A549 cells; 10^−4^ M∼10^−10^ M for PC-9 cells) alone significantly inhibited the proliferation of A549 and PC-9 cells in a dose-dependent manner (*P*<0.05). The IC_50_ of docetaxel and gefitinib were 2.05×10^−7^ mol/L and 1.22×10^−5^ mol/L respe ctively for A549 cells (Fig. 1A), and were 2.41×10^−8 ^mol/L and 5.65×10^−8 ^mol/L respectively for PC-9 cells (Fig. 1B). In particular, the inhibitory effect of gefitinib on PC-9 cells was almost 10^3^-folds stronger than that on A549 cells (*P*<0.05).

**Figure 2:**
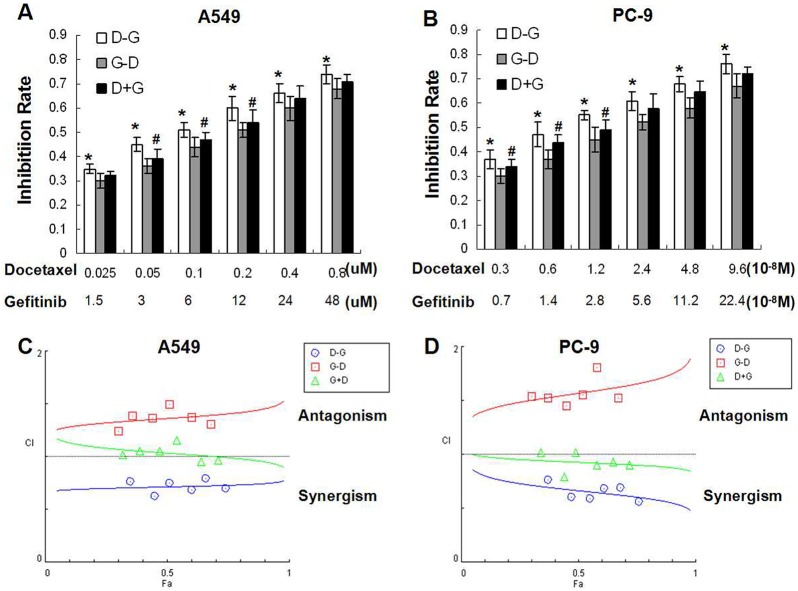
Effects of different exposure schedules of gefitinib and docetaxel on cell proliferation. Cells were treated with three different sequences of gefitinib and docetaxel (D→G; G→D; D+G). The drug doses were combined using constant ratios of IC50 values (0.125, 0.25, 0.5, 1, 2 and 4 times of IC50). (**A, B**) The inhibition rate was determined by MTT assay. *P<0.05, D→G versus G→D; #*P*<0.05 D→G versus D+G. The D→G sequence produced the most potent inhibitory effect. (**C, D**) The combination index (CI) was calculated using CompuSyn software. Only the D→G sequence showed synergistic effect. (D–G) docetaxel followed by gefitinib; (G–D) gefitinib followed by docetaxel; (D+G) docetaxel and gefitinib administered concurrently. *Bars*: ± SD, n = 3.

We then evaluated the antiproliferative effects of different exposure schedules of gefitinib and docetaxel on both EGFR-TKI-resistant (A549) and EGFR-TKI-sensitive (PC-9) cell lines. As shown in figure 2, only sequential administration of docetaxel followed by gefitinib (D→G) induced a clear synergistic effect (CI<0.9) in both cell lines (inhibitory effects at IC_50_ were 60.00±4.90% for A549 and 62.15±3.84% for PC-9 cells). On the contrary, the G→D sequence resulted in an antagonistic interaction (CI>1.1) in both cell lines. Concurrent administration of docetaxel and gefitinib (D+G) showed antagonistic and additive (0.9<CI<1.1) effects in A549 cells as the drug concentration increased; whereas in PC-9 cells, D+G showed additive effects in low dose combination and synergistic effects in high dose combination.

### Effects of different exposure schedules of gefitinib and docetaxel on cell signaling pathways

**Figure 3:**
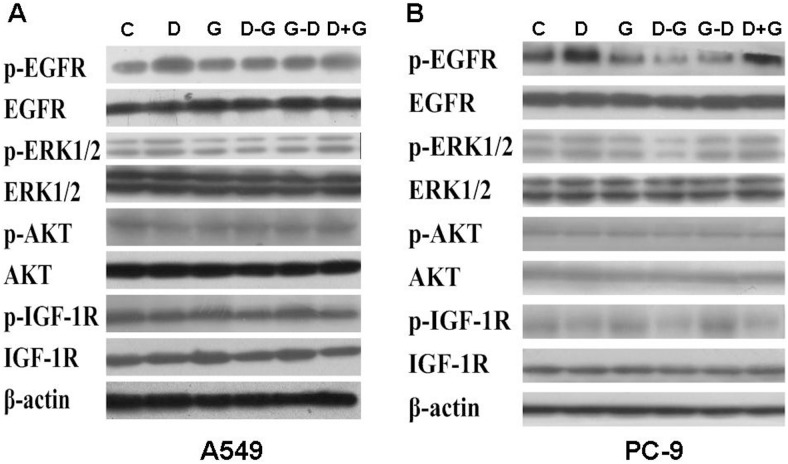
Effects of different exposure schedules of gefitinib and docetaxel on cell signaling pathways. The expression and phosphorylation of some representative molecules in correlated cell signaling pathways including EGFR, ERK, Akt and IGF-1R were detected by western blot. (**A**) A549 cells; (**B**) PC-9 cells. (C) control group; (D) docetaxel alone; (G) gefitinib alone; (D–G) docetaxel followed by gefitinib; (G–D) gefitinib followed by docetaxel; (D+G) docetaxel and gefitinib administered concurrently.

We further probed into the possible mechanism of the sequence-dependent effect of gefitinib and docetaxel. Figure 3 showed that for both cell lines, docetaxel alone enhanced the phosphorylation of EGFR and ERK, whereas gefitinib alone repressed the phosphorylation of these two proteins. For D→G schedule, docetaxel-enhanced phosphorylation of EGFR and ERK was significantly repressed by subsequently used gefitinib, and this reverse effect was much stronger in EGFR-mutant PC-9 cells. On the contrary, in regard to G+D and G→D schedules, gefitinib-repressed phosphorylation of EGFR and ERK was reversed by neither concurrent nor subsequent administration of docetaxel.

Both D→G and D+G schedules significantly inhibited the phosphorylation of IGF-1R in PC-9 cells, whereas in A549 cells, such inhibition effect was barely detected. On the contrary, G→D schedule increased the phosphorylation of IGF-1R in both cell lines. Besides, none of the three schedules showed detectable effect on the expression and phosphorylation of AKT.

### Effects of different schedules of gefitinib and docetaxel on cell cycle distritution

**Figure 4:**
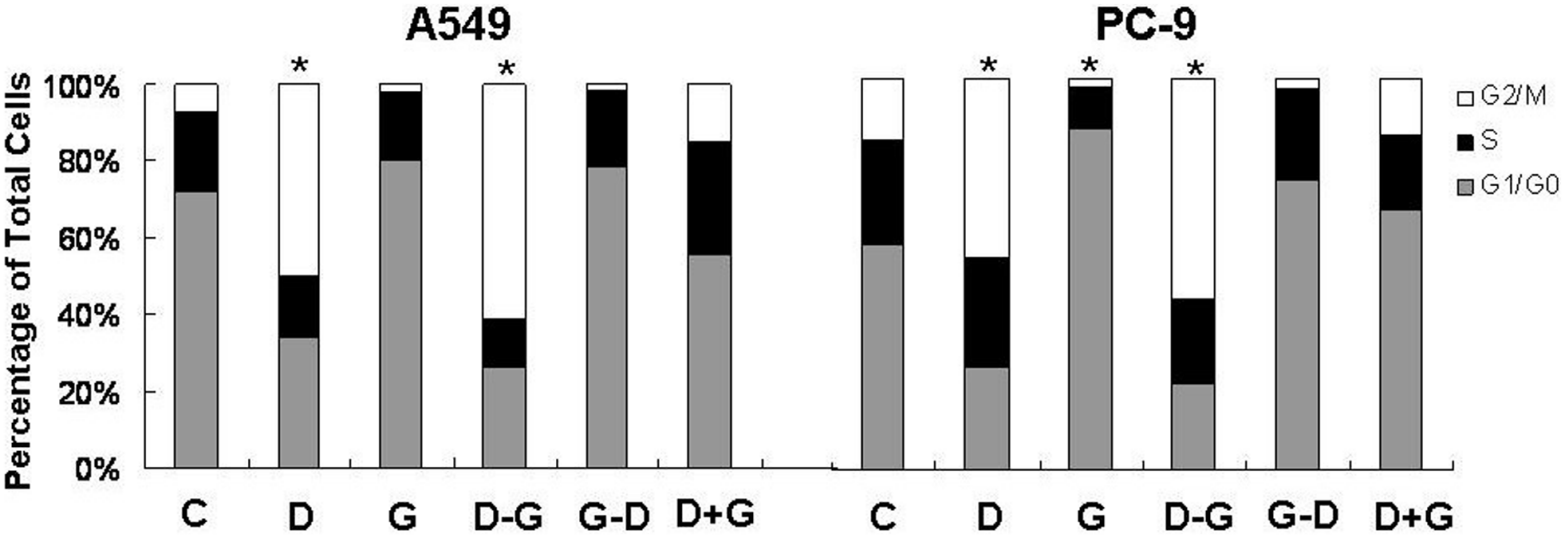
Effects of different exposure schedules of gefitinib and docetaxel on cell cycle distribution. The effects of different administration schedules of gefitinib and docetaxel on cell cycle distribution were detected by flow cytometry. (C) control group; (D) docetaxel alone; (G) gefitinib alone; (D–G) docetaxel followed by gefitinib; (G–D) gefitinib followed by docetaxel; (D+G) docetaxel and gefitinib administered concurrently. *P<0.05 compared with control group. n = 3.

Figure 4 showed that for PC-9 cells, gefitinib alone induced significant G_0_/G_1_ phase arrest comparing to control group (86.94±2.33% vs. 57.32±3.79%, *P*<0.05); while docetaxel alone induced significant G_2_/M phase arrest (45.67±3.90% vs. 15.54±2.57%, *P*<0.05). D→G also significantly arrested the cell cycle in G_2_/M phase (56.31±1.86% vs. 15.54±2.57%, *P*<0.05). However, G→D showed merely a trend of increased G_0_/G_1_ phase (74.39±2.78% vs. 57.32±3.79%, *P>*0.05).

For A549 cells, gefitinib alone had no remarkable effects on cell cycle distribution, while docetaxel alone induced significant G_2_/M phase arrest comparing to control group (49.58±2.70% vs. 6.87±1.35%, *P*<0.05). D→G also significantly arrested the cell cycle in G_2_/M phase (60.74±3.57% vs. 6.87±1.35%, *P*<0.05). However, G→D showed merely a trend of increased G_0_/G_1_ phase (78.42±2.52% vs. 72.13±3.89%, *P>*0.05).

## Discussion

The present study investigated the sequence-dependent antiproliferative effects of gefitinib and docetaxel in three different combination schedules in both PC-9 cells (EGFR mutant, K-ras WT) and A549 cells (EGFR WT, K-ras mutant). When gefitinib and docetaxel were used alone in the two cell lines, the inhibitory effect of gefitinib on PC-9 cells was dramatically stronger than that on A549 cells, which was in accordance with previous reports [1]. However, the IC50 of gefitinib was only half in PC-9 compared with that in A549; while in clinical work, gefitinib is far more effective for patients with EGFR mutation than docetaxel. We supposed this might be because in *in* *vitro* study, the testing agents were applied directly on target cells, but in *in* *vivo* study the agents were usually given through circulation system, which could affect the distribution and concentration of different agents.

Our results also demonstrated that docetaxel followed by gefitinib (D→G) was significantly superior to other modes in regard to the anti-tumor effects not only in EGFR mutant and K-ras WT PC-9 cells, but also in EGFR WT and K-ras mutant A549 cells. This was in accordance with several preclinical studies which enrolled a panel of different lung cancer cell lines treated with chemotherapy/EGFR-TKI and reported that the sequence of chemotherapy→EGFR-TKI had advantage over other modalities [8], [9], [15]. Some phase III clinical trials including WJTOG0203, INFORM and FASTACT-2 also validated that the sequential administration of chemotherapy followed by EGFR-TKI remarkably improved the outcome [4]–[6]. Therefore our findings produced strong preclinical support for surmounting the therapeutic plateau of EGFR-TKI and chemotherapy, and might be especially important for combating against refractory NSCLC. It is needed to be noted that in the present study, the D→G synergism was also observed in EGFR wild-type cell lines. Though similar results were also reported by some preclinical and clinical trials [8], [16], [17], many more data indicated that patients with wild-type EGFR could not benefit from EGFR-TKI treatment [4]–[6]. We presumed that it was because in our study IC50 doses of gefitnib for each cell line were applied, which meant a thousand folds of gefitinib was used for A549 cells compared with PC-9 cells. Obviously such extremely high doses could not be duplicated directly in clinical work, but relatively high-dose of EGFR-TKI has already been tried for NSCLC patients with brain metastases and positive results were reported [18], [19]. It should be especially beneficial for patients with wild-type EGFR if similar modes were tried and succeeded in such subgroup.

In regard to concurrent administration of docetaxel and gefitinib, our result seemed kind of complicated, with additive (0.9<CI<1.1)-synergistic effect observed in PC-9 cells and antagonistic-additive effect in A549 cells. This indicated that D+G was more effective in EGFR mutant cells than in wild-type cells. Such data corresponded to other preclinical reports which indicated that EGFR gene status might be a predictor for the activity of simultaneous combination [9]. Though early phase III trials failed to show advantage of concurrent combination of chemotherapy + EGFR-TKI over chemotherapy alone in unselected patients, a subgroup analysis of TRIBUTE demonstrated that erlotinib concurrently combined with chemotherapy conferred a remarkably higher response rate in patients with mutant EGFR than patients with wild-type EGFR (53% vs. 18%, *P*<0.01) [20]. Therefore we presumed that for concurrent combination, EGFR mutation might predict a therapeutic benefit. Similarly, it is also needed to be noted that the doses of gefitinib used in A549 cells was much more higher than that in PC-9 cells, which might have leaded to the D+G -induced additive effect observed in A549 cells.

We further probed into the mechanism underlying the synergism induced by D→G schedule. It has been demonstrated that EGFR-TKIs competitively bound with EGFR and blocked several downstream signaling pathways including PI3K/Akt and MARK/Erk, resulted in inhibition of tumor cell proliferation, tumor vascularization and metastasis [21], [22]. A schedule study from Jiang et al. revealed that the synergism of D→G schedule might be related with MAPK phosphorylation ratio [15]. Our data showed that the phosphorylation of EGFR and ERK (pEGFR and pERK) was enhanced by docetaxel and was suppressed by gefitinib. When gefitinib was used after docetaxel (D→G), docetaxel-increased pEGFR and pERK was repressed by subsequently used gefitinib. However, this reversal was not detected in other two schedules. This was in accordance with Giovannetti’s [8] and Van Schaeybroeck’s [23] studies which reported that only those cells exhibited increased pEGFR could benefit from sequentially used EGFR-TKI. Therefore it was suggested that chemotherapy-induced up-regulation of pEGFR and pERK reinforced the sensitivity of lung cancer cells to subsequently used EGFR-TKI, and finally resulted in improved outcomes.

Western blot also showed that gefitinib-induced repression of pEGFR and pERK was not reversed by concurrent exposure of docetaxel, it is therefore assumed that gefitinib failed to reinforce the inhibitory effect of simultaneously used docetaxel due to the low level of pEGFR and pERK in A549 cells. Consistently, Van Schaeybroeck et al. [24] demonstrated that in colorectal cancer cells, down-regulation of pEGFR led to the antagonistic interaction between EGFR-TKI and cytotoxic agents. On the contrary, synergistic activity of simultaneous administration was observed in EGFR mutant cells PC-9. However, according to our results mentioned above, EGFR and ERK phosphorylation might not be the possible mechanism. There were multiple evidences showing that IGF-1R expression was correlated with cell sensitivity to chemotherapy and EGFR-TKIs [25], [26]. Interference of IGF-1R expression or the administration of IGF-1R inhibitor improved the outcome of cytotoxic agents and EGFR-TKIs, which might be correlated with the suppression of PI3K/Akt pathway [27], [28]. In the present study, D+G significantly inhibited the phosphorylation of IGF-1R in PC-9 cells, whereas in A549 cells, such inhibition effect was barely detected. We therefore assumed that the strong inhibition of IGF-1R phosphorylation might contribute to the synergism achieved in PC-9 cells. Further confirmation is warranted by up-regulation and down-regulation of pIGF-1R in PC-9 cells.

The G→D schedule showed antagonistic effect in both A549 and PC-9 cells. However, the regulation of pEGFR and pERK failed to provide a plausible explanation as gefitinib-induced down-regulation of pEGFR and pERK was not reversed by subsequent exposure to docetaxel. In accordance with other reseachers’ previous studies [29], the flow cytometry analysis demonstrated that gefitinib decreased cells in S and G_2_/M phase. Such decrease of cells in proliferative phase might attenuate the cell sensitivity to subsequently used docetaxel which selectively targeted at the G_2_/M phase cells, and result in antagonistic effects.

## Conclusion

Collectively, our data suggested that among the combination modalities available in clinical work, the most effective approach was the sequence of using cytotoxic agents followed by EGFR-TKI. The phosphorylation of EGFR and ERK might have contributed to this synergy. However, since only two cell lines were enrolled in this study, expansion to a panel of NSCLC cell lines with different EGFR status as well as the *in* *vivo* studies on animal models are warranted to further assess the schedule-dependent effect. It is also worth comparing the three combination schedules in clinical trials to identify the possible “optimal” treatment modality for patients with advanced NSCLC.
